# TV medical dramas: health sciences students’ viewing habits and potential for teaching issues related to bioethics and professionalism

**DOI:** 10.1186/s12909-021-02947-7

**Published:** 2021-09-26

**Authors:** Irene Cambra-Badii, Elisabet Moyano, Irene Ortega, Josep-E Baños, Mariano Sentí

**Affiliations:** 1 Chair in Bioethics, Universitat de Vic – Universitat Central de Catalunya, Carrer Miquel Marti i Pol, 1, 08500 Vic, Spain; 2 Research Group Education in Health Sciences, Department of Experimental and Health Sciences, Universitat Pompeu Fabra, Barcelona, Spain; 3 School of Health and Life Sciences, Universitat Pompeu Fabra, Barcelona, Spain; 4 School of Medicine, Universitat de Vic – Universitat Central de Catalunya, Vic, Spain

**Keywords:** Bioethics, Professionalism, Health sciences students, Television, Medical Dramas

## Abstract

**Background:**

Medical dramas have been popular since their inception, especially among medical students. We hypothesized that the recent increase in the availability of TV medical series through online streaming platforms has probably changed health science students’ viewing habits as well as the representation of bioethical conflicts and health professionals.

**Methods:**

We invited undergraduate students of medicine, nursing, and human biology to complete a self-administered questionnaire about their viewing habits and perceptions of the depictions of bioethical issues and professionalism in TV medical series.

**Results:**

Of the 355 respondents, 98.6 % had watched TV in the last year, 93.5 % watched TV series, and 49.6 % watched medical dramas more than once a week. The most-viewed medical dramas were *The Good Doctor*, *House MD*, and *Grey’s Anatomy*. The most-remembered bioethical topics were medical errors, inappropriate professional behaviors, and death. Most students considered that ideals of professionalism were depicted positively and professionals were portrayed as intelligent, professionally qualified, and competent.

**Conclusions:**

Medical dramas are very popular with health science students and are potentially useful as teaching tools for discussing issues related to bioethics and professionalism.

**Supplementary Information:**

The online version contains supplementary material available at 10.1186/s12909-021-02947-7.

## Background

TV medical dramas have been very popular since their inception [1–5]. Although most TV medical dramas are produced in the United States and depict situations in the American health system, they are consumed globally. Medical dramas are mainly watched for entertainment and relaxation [6]; they are followed by many medical and nursing students [7–10] as well as by clinicians [11].

These fictional narratives provide concrete medical situations and are often regarded as a source of health information by viewers [1, 12]. Several authors have suggested that medical dramas are a potential tool for teaching students in health sciences disciplines [2, 5, 13, 14]. Baños et al. [15] analyzed the usefulness of *House MD* for teaching clinical pharmacology. Jerrentrup et al. [3] also proposed *House MD* to teach about rare diseases and diagnostic strategies. Williams et al. [16] pointed out several potential uses of *House MD*, *Scrubs*, and *Grey’s Anatomy* to teach medical problems, medical system issues, psychosocial issues, and relationships with the patient’s family. Wong et al. [17] successfully used two excerpts from *House MD* and one from *Grey´s Anatomy* to teach doctor-patient communication skills, while McNeilly and Wengel [18] used *ER* for teaching psychotherapeutic techniques to medical students. Hirt et al. [19] suggested *Northern Exposure*, *Cardiac Arrest*, *ER*, *Scrubs*, *House MD*, *Doc Martin*, *Grey’s Anatomy*, and *Nurse Jackie* in a brief guide for health sciences educators to teach various themes such as mentorship, hospital environments, teaching and learning, and professionalism.

Specifically for analyzing and teaching professionalism and bioethical issues, medical dramas were especially explored in the last decade. Czarny et al. [20] performed a content analysis of 50 episodes of *Grey’s Anatomy* and *House MD* during the 2005–2006 television season and found 79 depictions of bioethical issues classified under 11 topics, including consent, ethically questionable departures from standard practice, death and dying, and confidentiality. Cambra Badii et al. [21] performed a content analysis of the first season of *The Good Doctor* and found 179 situations that can be used to teach bioethics. Arawi [22] proposed to work with *Grey’s Anatomy* and *House MD* vignettes to teach biomedical ethics, and Pavlov and Dalquist [23] used *Grey’s Anatomy* to teach communication and professionalism. Moreover, Spike [24] pointed out the bioethical issues involved in a doctor–patient communication and medical errors in *Scrubs*.

The portrayals of health professionals that appear in medical dramas are also interesting, because they were changing over the years [5, 14, 25]. Chory-Assad and Tamborini [26, 27] demonstrated the decline in positive portrayals of physicians in television since the 1990 s, and Czarny et al. [20] showed up clearly anomalous portrayals of professionalism in *House MD* and *Grey’s Anatomy*.

These portrayals can be used to analyze or discuss professionalism because they offers positive models of behavior, like Miranda Bailey and Derek Sheperd from *Grey’s Anatomy*, or Allison Cameron and James Wilson from *House MD* [9]. Even complex and negative models, like Doctor House in *House MD*, can be useful to teach professionalism, but in this case, from his mistakes [9, 20]. Seminal studies of television viewing habits of health sciences students have provided interesting information about health sciences students’ TV viewing habits [7, 9, 10]. However, in the last 10 years, the growing popularity of online platforms such as Netflix, HBO, Hulu, and Amazon Prime has drastically changed the way that TV series are produced and consumed [28]. It is likely that these developments have changed health sciences students’ TV viewing habits, and it is possible that they have also affected their impressions of bioethical issues and the portrayal of healthcare professionals in medical series.

Taking into account the previous literature and the expansion of series and online platforms, we hypothesized that health sciences students watch medical dramas almost daily, that they remember ethical dilemmas and how they were portrayed, and that they follow positive and negative role models, so medical dramas might therefore be useful for teaching them bioethics and professionalism. In this study, we sought to determine (a) health science students’ television viewing habits, (b) which medical dramas they have recently viewed, (c) their motives for watching medical dramas, (d) the ethical issues portrayed in the dramas that they recall, and (e) the characteristics of the health professionals’ portrayed in the dramas that determine whether students’ consider them positive or negative role models.

## Methods

### Study population

This study surveyed all undergraduate students of medicine, nursing, and human biology at our University during the 2018–2019 academic year. Inclusion criteria were participation in classroom activities at the time of the survey and consent to participate in the study. Exclusion criteria were not explicitly established other than the lack of compliance with inclusion criteria. The final sample population included all students enrolled in the 6-year Bachelor of Medicine program, in the first 3 years of the 4-year Bachelor in Nursing program, and in all years of the 4-year Bachelor in Human Biology. Fourth-year nursing students were not included because the fourth year of this program is dedicated to clinical training and does not include classroom activities. Medical students begin clinical training in the fourth year, but continue to have classroom activities thereafter, and students of human biology do not undergo clinical training.

### Study design

We adapted Czarny’s medical TV drama survey [10], designed to gather data on basic demographic characteristics, general TV and TV medical drama viewing habits, impressions of bioethical and professionalism issues in medical dramas, and sources of information on bioethical issues (Additional file 1).

Medical dramas considered in the study were *ER*, *House MD*, *Grey’s Anatomy*, *NipTuck* and *Scrubs* like in previous studies [7, 9, 10]. We have added the most newest medical dramas aired in Spain at the time of the survey, *The Good Doctor* and *The Resident*. Students could add other medical dramas in an open field. We modified some questions about TV shows and the characters that students most and least wish to resemble in their professional lives to make them open-ended and adding some questions about the reasons for watching medical series [6] and the portrayal of doctors and nurses in medical dramas [26].

To ascertain whether the wording or the questions should be changed, we pilot-tested the survey on ten members of the research team. They were experienced researchers in the field of biomedical research education and members of the Group for Educative Research in Health Sciences of our university. Based on this feedback, we reformatted the survey to improve clarity.

### Data collection

We used the Google Forms platform to draft the questionnaire and conduct the survey; participants could complete the questionnaire between February 13 and April 8, 2019. We ensured that each respondent provided only one response by controlling the login in Google Forms in the different classrooms, while guaranteeing anonymity.

The study was performed according to the principles of the Declaration of Helsinki. Data confidentiality was ensured according to local legislation on the protection of personal data.

Before starting the survey, students were read an information sheet explaining the background, aims, and procedure of the study, and they were informed that the bioethics committee had approved the study protocol and that participation was voluntary.

### Data analysis

We used descriptive statistics to summarize participants’ demographic characteristics and data obtained from the survey. To compare categorical variables between groups (men vs. women; medical students vs. nursing students vs. human biology students; preclinical vs. clinical medical students), we used chi-square tests.

In analyzing our data, we dichotomized responses for three items. For the frequency of viewing medical series, we grouped all responses indicating viewing > 1 under the category “frequently” [9]. For questions answered on a five-item Likert scale (those regarding the reasons for viewing medical dramas and the representation of ethical issues), the responses “totally agree” and “agree” were grouped together into the category “yes”, and the responses “neither agree nor disagree”, “disagree”, and “strongly disagree” were grouped into the category “no”. Similarly, the responses about the appropriateness of the depiction of each of the different ethical issues in the medical series, the responses “barely” and “below acceptable” were grouped together into the category “poorly represented”, while the responses “appropriate” and “very appropriate” were grouped together as “well represented”.

All tests were two-tailed, and statistical significance was set at 0.05. Microsoft Office Excel 2007 and SPSS (version 16) were used for all analyses.

## Results

### Study population

A total of 355 of 918 students participated in the survey; responses were obtained from 124/339 (36.6 %) of eligible medical students, 143/381 (37.5 %) of eligible nursing students, and 88/198 (44.4 %) of eligible human biology students (Table 1). Women accounted for 78.5 % of the respondents, and the proportion of women participants was similar in the three programs, coinciding with the distribution of women in the three programs. Mean age did not differ between groups of students from the different programs, although the age range was broader in the group of nursing students than in the other two groups.

**Table 1:** Characteristics of the study sample

Undergraduate program	Gender, n (%)		Age, mean (SD)	Year, mean (SD)
	M	F		
Medicine	36 (29.5)	86 (70.5)	22.1 (0.4)	3.8 (1.7)
Nursing	21 (14.7)	122 (85.3)	21.0 (3.4)	1.8 (0.8)
Human Biology	19 (21.6)	69 (78.4)	19.8 (0.2)	2.4 (1.1)

### Television viewing habits

Nearly all respondents (98.6 %) reported watching TV in the last year; the most-watched shows were TV series (93.5 %), followed by movies (86.8 %), news (67.9 %), and sports (34.7 %) (Table 2). There were no differences between genders except women in the nursing program watched less news and sports (*p* < 0.05).

**Table 2:** Health science students’ TV viewing

Academic program	Watch TV		Type of TV program								Watch medical dramas			Format				Frequency (>1 time p/week)
			Series		Movies		News		Sports		Total			Regular episodes on TV	Reruns on TV	DVD	Online platforms	
Medicine	M 35 (97.2)	F 86 (100)	M 30 (83.3)	F 83 (9.5)	M 34 (94.4)	F 76 (88.4)	M 29 (8.6)	F 68 (79.1)	M 18 (50)	F 30 (3.9)	57 (46)	M 12 (33.3)	F 45 (52.3)*	10 (17.5)	15 (26.3)	1 (1.8)	54 **	36 (29)
Nursing	M 20 (95.2)	F 122 (100)	M 20 (95.2)	F 115 (94.3)	M 17 (81)	F 96 (78.7)	M 17 (81)	F 66 (54)*	M 13 (61.9)	F 29 (23.8)*	80 (55.9)	M 6 (28.6)	F 74 (70.7)*	32 (40)	13 (16.3)	1 (1,3)	94**	78 (54.5)^$^
Human Biology	M 18 (94.7)	F 68 (98.6)	M 18 (94.7)	F 65 (94.2)	M 19 (100)	F 65 (94.2)	M 15 (78.9)	F 45 (65.2)	M 10 (52.6)	F 22 (32.4)	39 (44.3)	M 3 (15.8)	F 36 (52.2)*	13 (33.3)	12 (30.8)	--	42**	25 (28.4)

Data are expressed as number of cases and percentages in parentheses.

^*^Significantly different between genders, *p* < 0.05

^**^Significantly different from other formats, *p* < 0.05

^$^Significantly different from other academic programs

Nearly half (49.6 %) the students watched medical dramas. There were no differences in the frequency of viewing among the different programs.

The most common way of watching medical dramas was through online platforms (listed by 78.1 % of respondents: of whom 41.7 % used Netflix, 23.7 % used Movistar, 9.9 % used HBO, and 2.8 % used Amazon), followed by regular TV episodes (24.2 %), regular TV reruns (16.3 %), pirate streaming (11.5 %), and DVD (1.4 %).

In the last year, the most-watched medical dramas were *The Good Doctor* (47 %), *House MD* (41.4 %), and *Grey’s Anatomy* (38 %), followed by *The Resident* (7.9 %), *ER* (6.8 %), and *Scrubs* (5.9 %). No series included in the open field “other medical series” reached a significant frequency to be included in the analysis, but *Chicago Med*, *Code Black* and *Nurse Jackie* were mentioned a total of 16 times.

Gender differences were observed only for *Grey’s Anatomy*, watched by 44.7 % of women but only 13.1 % of men. The difference was even greater among medical students (46.5 % of women vs. 8.3 % men, *p* < 0.001). No differences were found in the medical dramas watched between students’ in different years within academic programs.

Asked whether they discuss medical aspects of the series with their friends, 57.8 % of all participants responded affirmatively. The reasons most often given for watching medical dramas were entertainment (96.6 %) and medical information (89.2 %), followed by habitual pastime/companionship (76.5 %), and relaxation (68 %).

### Ethical issues

Most students (56.9 %) considered that the medical series did not adequately portray the bioethical issues that appear in clinical practice. The bioethical issues remembered best were medical errors (66.5 %), inappropriate professional behavior (58.3 %), and death (59.4 %), followed by confidentiality (49 %), quality or value of life (48.2 %), infectious diseases (45.9 %), and access to and equity in health care (40.8 %). The least-mentioned issues were harassment (29.3 %), education for healthcare professionals (25.6 %), non-therapeutic methods (24.8 %), and human research (22.3 %) (Table 3).

**Table 3:** Recall of ethical issues in TV medical dramas

	Access to and equity in health care		Medical errors		Infectious diseases		Quality / Value of life		Non-therapeutic methods		Education for healthcare professionals		Informed consent		Human research		Artificial and transplanted organs / tissues		Death and dying		Discrimination		Harassment	
	M	F	M	F	M	F	M	F	M	F	M	F	M	F	M	F	M	F	M	F	M	F	M	F
Medicine	52.8	50	61.1	69.8	50	47.7	38.9	41.9	30.6	25.6	30.6	30.2	33.3	44.2	13.9	23.3	38.9	40.7	52.8	60.5	44.4	40.7	33.3	26.7
Nursing	33.3*	32	66.7	68.9	52.4	42.6	52.4	53.3	38.1	22.1	33.3	25.5	28.6	40.2	42.9	19.7	47.6	48.4	57.1	64.8	38.1	48.4	33.3	30.3
Human Biology	47.4	37.7	47.4	66.7	31.6	50.7	42.1	52.2	15.8	24.6	15.8	26.1	42.1	40.6	21.1	24.6	42.1	44.9	36.8	58	36.8	33.3	26.3	27.5

Data are expressed as percentages

^*^Significantly different from other academic programs, *P* < 0.05

The ethical issues that were considered the best portrayed in the medical dramas differed among the groups. Medical students most often listed death (65.9 %), infectious diseases (62.7 %), and quality or value of life (62.1 %); nursing students most often listed organ transplantation (67 %), death (62.5 %), and quality or value of life (59.3 %); and human biology students most often listed death (84 %), infectious diseases (81.6 %), and organ transplantation (80.9 %).

Similarly, the ethical issues considered badly represented listed by medical students were non-therapeutic medical uses (65.1 %), human research (64.3 %), and education of health professionals (58.8 %); those listed by nursing students were human research (64.1 %), access to and equity in health care (63.2 %), and non-therapeutic medical uses (62.5 %); and those listed by human biology students were access to and equity in health care (65.9 %), human research (59.5 %), and harassment (51.1 %).

There were no significant differences between clinical and preclinical medical students regarding the representation of bioethical issues: 45.2 % of preclinical students and 54.8 % of clinical students responded that these issues were well represented.

### Sources of bioethical information

The information about bioethics that students received during their university education came mostly in compulsory subjects (52.4 %), followed by extracurricular activities (5.1 %), and elective subjects (2.3 %); 15.5 % of respondents stated that they had received no information from the university about bioethical issues.

The sources of information on bioethics considered most important were the university (91.3 %) and scientific journals (86.8 %), followed by friends (76.4 %) and medical dramas (63.6 %). The sources considered less important were ecclesiastical staff, religious values, ​​and blogs. There were no differences between genders. There were no differences between fields of study, except medical students gave significantly lower importance to scientific journals and religious values.

### Portrayals of health professionals

Most students believed that all ideals of professionalism were depicted positively in medical dramas. Doctors were portrayed positively in medical dramas; respondents referred to their intelligence (97.3 %), professional qualification (93.6 %), and competence (90.5 %). Nurses were also portrayed positively; respondents most often referred to their kindness (75.8 %), empathy (74.5 %), and caring (74.3 %).

The survey asked about which characters the students aspire to be like in their professional careers, and they responded none (9.9 %), Dr. Meredith Grey (from *Grey’s Anatomy*) (5.6 %), and Dr. Gregory House (from *House MD*) (2.5 %). Students of human biology tended to want to resemble Dr. House more than medical students and nurses.

The characters they least aspired to be like were Dr. Gregory House (from *House MD*) (18.6 %) and Cristina Yang (1.4 %) and April Kepner (1.1 %) (both from *Grey’s Anatomy*). The most-chosen negative characteristics were lack of intelligence (55.1 %), lack of professional qualification (53.1 %), and lack of competence (42.2 %).

## Discussion

More than 12 years after Czarny et al.’s original survey of health science students’ TV viewing habits [10], we found that nearly all health sciences students had watched TV in the last year, corroborating the findings of other studies in the interim [7, 9]. Moreover, TV medical dramas were the type of program that students’ watched most. This is a notable difference with Weaver and Wilson’s study [9], where medical dramas were the least-watched category (films were the most-watched category). This difference could be explained by the increased number of TV series and their availability through online platforms (e.g., Netflix); students now have greater freedom to choose what they want to see.

Nevertheless, the percentage of students who said they watch medical series (49.6 %) was much lower than in earlier studies (> 80 % in all), although we asked specifically about medical dramas, which can be considered a subgenre of medical programs. We cannot know whether these findings represent a change of health science students’ interests or whether they are a consequence of the proliferation of programs and series in various genres.

The medical drama that health science students watched most was *The Good Doctor*, one of the newest medical dramas (available since 2017). This series shows the life of a young autistic physician with savant syndrome who is starting his residency in surgery, and it includes many situations that involve bioethical issues [21]. *The Good Doctor* approaches medical issues from a different point of view than other popular medical dramas, and the originality of using an autistic resident as the main character probably adds to its appeal.

Two of the most-viewed series in previous surveys were also among the most popular in the current study: *House MD*, which aired from 2008 to 2012, and *Grey’s Anatomy*, which has aired from 2005 and is now in its 16th season. Why do students continue to watch these TV series? *House MD* is considered one of the best medical dramas with an iconic protagonist [3, 7–10, 17, 29] and, like *Grey’s Anatomy*, it is distributed by Netflix, one of the most popular media-services providers, with 193 million subscribers around the world in 2020 [30]. Our study also corroborates the findings in previous research [9, 10] that *Grey’s Anatomy* was the only medical drama for which there were differences in viewing between the sexes. A greater proportion of women than men watched this medical drama, perhaps because the main character is a woman and the storyline revolves around social and love relationships and the daily challenges of clinical practice in a hospital [13]. Nevertheless, the percentage of women who regularly follow *Grey’s Anatomy* in our survey (44.7 %) is clearly lower than in Czarny’s study (81 %) but close to and Weaver and Wilson’s (48.2 %) survey. The drop in the consumption of this medical drama could be due to the age of the series (it debuted in 2005) as well as to the wide availability of competing offerings. The series *ER* and *Scrubs*, found to be popular in previous research [7–9], were not among the most-watched programs in the present study, probably because they are not currently available on online viewing platforms.

The most important reasons for viewing medical dramas in our study were different from those in Lee and Taylor’s factor analysis of data from students enrolled in communications courses [6], where social interaction, relaxation, and entertainment motives were significant predictors of viewing time. In the current study, the salient motives were entertainment and medical information. The sources of information on bioethical issues considered most important in the current study are in line with those reported in previous studies [7, 10], underlining the importance of the university and scientific journals. However, in the second line, friendships have replaced family. Medical dramas continued to hold the third place, as in Williams’ [7] and Czarny’s [10] studies, although it is interesting to note that whereas less than 50 % of respondents in their studies considered medical dramas an important source of information about bioethical issues, more than 60 % of those in our study considered these programs important.

Over half the respondents in our study considered that the ethical issues that appear in medical dramas were not adequately depicted. We can only speculate about the reasons for this finding, which is similar to that reported in previous investigations [7, 9, 10], although the study populations were different. Perhaps students consider that medical dramas are unrealistic. It is important to note that students recall the ethical issues portrayed in medical dramas even some time after viewing and consider that they are not correctly represented. Given that nearly half the students discuss medical issues with friends and that “social activity may facilitate the formation of new opinions and perceptions” [10], it seems that medical dramas play a role in forming views about bioethics and appropriate behavior for health professionals.

We analyzed the portrayal of medical doctors and nurses in medical dramas and the characteristics of these professionals that the students consider positive. Both professions were viewed positively. The characters in medical dramas have changed over the last 25 years, evolving from idealized representations of “medical heroes” to more complex representations of professionals with flaws and interpersonal problems, even to the point of including “antiheroes” [25–27, 31]. These changes seem to point to an increased focus on negative characteristics of physicians in current medical dramas; as Chory-Assad and Tamborrini [26, 27] demonstrated, positive portrayals of physicians in television programs have declined since the 1990 s. However, students of health sciences continue to consider that the protagonists of these TV series are positively represented.

It is striking that we found the least-represented characteristics of the physicians in medical dramas were empathy, emotional involvement, and kindness. Our results agree partially with those of Chory-Assad and Tamborrini’s earlier studies [26, 27] although their analyses included the representation of doctors in different types of television programs, not only medical dramas. Like in our study, “competence” was an important characteristic of TV physicians in their studies; however, they found that “ethical character” and “regard for others” were also portrayed. These findings highlight a shift in emphasis toward knowledge derived from evidence-based medicine to the detriment of clinical skills needed to care for patients [32].

As in Weaver and Wilson’s study [9], the characters students would most like to resemble continue to be the protagonists of *House MD* and *Grey’s Anatomy*, the most-viewed medical dramas. Also, Dr. Gregory House ranked highly both as someone students most and least aspired to be like in their own careers. The importance of this character should not be underestimated: despite his questionable professionalism evident in his constant disregard for the rules and avoidance of contact with his patients [33, 34], health sciences students value House’s knowledge and intelligence [3, 29].

The characters that respondents from all three programs of study said they would most like to resemble were all medical doctors. The fact that respondents did not choose any nursing characters invites speculation about the roles of doctors and nurses in TV medical dramas. Whereas the characteristics most often cited to describe physicians in these series were intelligence, professional qualification, and competence, those most often cited to describe nurses were friendliness, empathy, and caring. These portrayals have been analyzed various times in relation with the different roles of physicians and nurses as well as with poor, incorrect, or misleading portrayals of nursing role models [8, 35] in which stereotypes abound, especially in relation to gender roles [36]. Nurses are rarely the protagonists of medical dramas and are even “invisible” in many TV dramas [37]; moreover, nurses are often wrongly characterized as only females and as underlings who are not involved in decision making.

Our results show that medical dramas could be useful for teaching bioethics. Because these programs are seen by many students, have an interesting and entertaining format, depict bioethical conflicts that students remember and characters they want to resemble, TV medical dramas can be considered a valuable tool to help students reflect on bioethics. Many TV series present diverse situations in each episode that can be used to teach ethics, although it is essential to analyze the content and to establish the suitability of the material for each pedagogical objective [38, 39].

Students’ interest in medical dramas suggests that these programs can also be considered in developing new teaching strategies. These materials can be useful for exploring transversal issues, such as medical professionalism, doctor-patient relationship, bioethics, and communication skills [2, 16, 19]. Medical dramas can also be considered part of the hidden and informal curricula of medical ethics [40–42], which is at least as important as formal education in ethics [10]. Given the importance of compulsory subjects in health sciences degrees in teaching of bioethics, incorporating medical dramas into these classes could be a good approach to exploiting this material.

Students can absorb the educational messages in medical dramas when they view them for entertainment. In fact, even though they were not created specifically for education, these programs can be seen as an entertainment-education tool [43, 44]. In entertainment-education shows, viewers are exposed to educational content in entertainment contexts, using visual language that is easy to understand and triggers emotional engagement [45]. The enhanced emotional engagement and cognitive development [5] and moral imagination make students more sensitive to training [22]. Previous investigations about entertainment-education medical dramas and social learning theory indicate that these programs can increase knowledge about health matters, for example about early breast cancer detection [46].

Albert Bandura’s social learning theory [47–49] indicates that people can learn behaviors vicariously by watching and thinking about characters’ behaviors. In entertainment-education medical drama, the characters can serve as positive or negative models to teach desirable professional behavior [9, 10].

Rather than merely passive observers, health science students are active viewers who notice positive and also negative characteristics of physicians and nurses. In this sense, students can decide whether characters’ behavior should be emulated or criticized, as well as which characters might serve as positive or negative role models. Also, students point out that bioethical dilemmas are sometimes poorly represented (overall, students most often mentioned inadequate representation of bioethical issues related to human research, non-therapeutic methods, and equity of access to healthcare) and this implies that they critically see portrayal of bioethical dilemmas and the character actions in resolving these dilemmas. As Spike points out [24], these TV series adhere to the Hollywood paradigm of morality tale and may be valuable aids to thinking about ethics and professionalism. Although it is difficult to determine the effects of medical dramas on students’ attitudes, our study confirms their popularity.

Some authors consider that TV series can have negative influences on students because they could be unrealistic or even potentially harmful and dangerous [34, 50], basically because of unreal or inaccurate depictions of hospital procedures and professional practice; however, we consider that rather than focusing on the authenticity of medical procedures, teaching based on medical series should focus on the plausibility of the situations that are presented [51]. The issues depicted in medical dramas are useful for exploring moral judgments beyond verisimilitude [29], although they may not be very useful for teaching practical skills [50] except to criticize mistakes. In any case, studies comparing the pedagogical efficacy of medical dramas and other narrative resources could provide valuable information.

Future research must apply a systematic approach to evaluate the pedagogical impact of medical dramas. Although there are some previous experiences [17, 18, 22], there is a lack of systematization in the development of these activities and in the measurements of empirical results; moreover, the approach to the pedagogical interpretation of this impact should also be considered systematically, for example using Kirkpatrick’s learning levels [52, 53].

The large number of health science students that watch TV series and medical dramas in particular strongly suggests that students find these programs attractive, which in turn makes them attractive for teaching If we interpret this association according to Kirkpatrick’s learning levels, it seems clear that the use of series in teaching of health sciences would easily fulfill the first level, of reaction. A positive reaction to the teaching activities is essential: although it does not guarantee learning by itself, it serves to validate the experience [52]. Future studies should evaluate teaching experiences through short- and long-term outcomes, measuring not only student satisfaction (Kirkpatrick’s first level), but also knowledge acquisition (Kirkpatrick’s second level) and even skills acquisition on the behavioral level (Kirkpatrick’s third level). This is especially important in relation to teaching bioethical issues that health science students already observe, remember, and criticize from medical series. These medical dramas could model behavior. It is striking that the most-remembered ethical issue was medical errors, followed by inappropriate professional behavior. As we pointed out above, these are opportunities to learn through good or bad models, so they can be included in classes to teach patient safety or other specific subjects with an appropriate evaluation method. We recommend strict measurement of the effectiveness of teaching activities as well as comparison with standard pedagogical methods.

As Law et al. [43] pointed out, it would be important to take into account long-term evaluations to address Kirkpatrick’s third (behavior) and fourth (results) learning levels. This approach involves not only measuring how medical dramas can lead to knowledge and skills acquisition, but also how this acquisition can be translated into students’ behavior in concrete situations, such as in workplace-based assessments or simulations about patient care, when they have to remember and reproduce for the action themselves.

Finally, some limitations of our study should be outlined. First, it was limited to a sample of health sciences students of some disciplines at a single university. Our study can be expanded in future research to include more students from the same university or students at various universities on different continents. Moreover, only about 50% of the students responded, probably because participation was voluntary, but we consider that was adequate to obtain sound conclusions. In fact, getting good responses to surveys is a challenging issue. Besides, the majority of respondents were women but this was expected as most of students of health sciences in our country are female.

It would be interesting to explore the effects of cognitive maturity on students’ responses; however, this important issue should be considered in a study specifically designed to determine the effect of cognitive maturity in each group of students. Also, it could be interesting to analyze the impact of local TV series to analyze bioethical issues outside the context of the United States [50, 54], and to analyze the impact of non-fictional programs. Moreover, it could be interesting to analyze the possible influence of medical dramas in young professionals’ attitudes and behaviors [8, 11] as well as the possible impact on young people’s desire to become healthcare professionals.

## Conclusions

We conclude that medical dramas are of considerable importance and relevance to students. Nearly all health sciences students watched television in the last year, and nearly half of them students watched medical dramas frequently. Students remember bioethical issues discussed in television series, and most students believed that all ideals of professionalism were depicted positively in medical dramas.

The results of the current study show the effects of the huge increase in the availability of TV medical series through online platforms on the viewing habits of health science students. These students are big consumers of TV series, especially on new platforms. These resources are very accessible, allowing students to watch episodes of current or previously broadcast series as often as they like and as many times as they like. Students can view them on various devices (televisions, computers, tablets, smart phones, etc.), alone or with their friends and families.

Medical dramas can be useful for teaching issues related to bioethical questions and professional practice in health sciences. Depending on their educational objectives, teaching initiatives could use an entire season, an episode, or a shorter edited clip. It is important to undertake new studies to analyze students’ viewing habits and educational proposals to exploit medical dramas. Information about students’ interests, the series they watch, the bioethical conflicts that they remember can help in designing teaching activities, and information about the efficacy of these adjuvant teaching methods can help in improving them.

## Supplementary Information

**Additional file 1. ** Television viewing habits survey

## Data Availability

The datasets used and/or analysed during the current study are available from the corresponding author on reasonable request.
