# Supplementary material for: TV medical dramas: health sciences students’ viewing habits and potential for teaching issues related to bioethics and professionalism
Source: BMC Med Educ. 2021 Sep 26;21:509. doi: 10.1186/s12909-021-02947-7 (PMC8474903; doi:10.1186/s12909-021-02947-7)
Supplement: Supplementary file 1 — Additional file 1. Television viewing habits survey [file 12909_2021_2947_MOESM1_ESM.docx]

# Appendix 1: Television viewing habits survey

1. Which program are you enrolled in?

Medicine

Nursing

Human Biology

2. What year are you currently in?

3. What is your age?

4. What is your gender?

5. Have you watched a television program in the last year? Yes / No

6. What types of program?

TV series

Movies

News

Sports

7. Do you usually follow medical dramas?

8. In the past year, have you watched any of the following?

ER

House MD

Grey’s Anatomy

NipTuck

Scrubs

The Good Doctor

The Resident

Other medical dramas

9. In what format?

Regular episodes on TV

TV reruns

DVD

Movistar online platform

HBO online platform

Neflix online platform

Other

10. How often?

Never

Less than once a week

1-3 times a week

4-6 times a week

More than 6 times a week

11. For those who answered the previous questions affirmatively: what are the reasons why you watch medical series? (Likert scale)

They make me think about health.

They inform me about diseases and treatments.

They tell me something about how I can maintain my health.

They help me understand how the health system works.

They help me understand some of the problems people have.

They let me see how other people live.

So I can be with my family or friends.

It is something to do when friends come over.

So I can talk with other people about what’s on TV.

Just because they are there.

They give me something to do to occupy my time.

They make me feel less lonely.

When there is no one else to talk or be with.

They entertain me.

They are exciting.

I like guessing the diagnosis when they admit patients and describe their symptoms.

They relax me.

They allow me to unwind.

12. Do you discuss the medical aspects of the series with your friends? Yes / No

13. Do you think that the medical dramas show the ethical issues that appear in clinical practice correctly? (Likert Scale)

14. What ethical issues do you remember from the television series?

Access to and equity in health care.

Medical errors.

Infectious diseases.

Quality / Value of life .

Non-therapeutic methods.

Education for healthcare professionals.

Informed consent.

Human research.

Artificial and transplanted organs / tissues.

Death and dying.

Discrimination.

Harassment.

15. Do you consider that, overall, they were adequately dealt with? (Likert scale on items of 14Q)

16. Did friends or family members ask you about your opinion of a bioethical issue in the show? Yes / No

17. Which of the following characteristics describe the doctors portrayed in these series?

Competent / Incompetent / Not applicable.

Caring and compassionate / Uncaring and lacking compassion / Not applicable.

Smart / Not smart / Not applicable.

Qualified professionally / Not qualified professionally / Not applicable.

Empathic / Lacking empathy / Not applicable.

Responsible / Irresponsible / Not applicable.

Emotionally involved with their patients / Emotionally detached from their patients / Not applicable.

Altruistic / Selfish / Not applicable.

Good / Bad / Not applicable.

Honest / Dishonest / Not applicable.

Moral / Immoral / Not applicable.

Warm / Cold / Not applicable.

Friendly / Unfriendly / Not applicable.

Physically attractive / Physically unattractive / Not applicable.

Kind / Unkind / Not applicable.

Anxious / Calm / Not applicable.

Rich / Poor / Not applicable.

Weak / Strong / Not applicable.

With personal problems / Without personal problems / Not applicable.

Respectful of the rules / Disrespectful of the rules / Not applicable.

18. Which of the following characteristics do the nurses portrayed in these series show? (same options as in question 17)

19. Which character in these medical series do you aspire to resemble in your professional career?

20 Why? (You can select all the options you want) (same options as in question 17)

21. What character in these medical series would you not want to resemble in your professional career?

22. Why? (You can select all the options you want) (same options as in question 17)

23. What is your university training in bioethics? None / Compulsory subjects / Elective subjects / Other activities

24. Please rank the following choices from 1 to 10 in terms of their importance in informing you about bioethical issues (where 1 is most important and 10 is the least important):

Friendships

Non-scientific magazines

University

Newspapers

Religious values

Scientific journals

Medical dramas

TV news

Blogs

Internet news

25. Is there anything else you want to comment on regarding the relationship between medical TV series and bioethics?
